# Near-Field Resonance Microwave Sounding to Study Dielectric Properties of Different Skin Areas (Experimental Study)

**DOI:** 10.17691/stm2020.12.5.06

**Published:** 2020-10-28

**Authors:** А.K. Martusevich, А.А. Epishkina, Е.S. Golygina, А.N. Tuzhilkin, А.S. Fedotova, А.G. Galka

**Affiliations:** Head of Medical Biophysics Laboratory, University Clinic; Privolzhsky Research Medical University, 10/1 Minin and Pozharsky Square, Nizhny Novgorod, 603950, Russia; Professor, Department of Animal Physiology and Biochemistry; Nizhny Novgorod State Agricultural Academy, 97 Prospekt Gagarina, Nizhny Novgorod, 603107;; PhD Student, Assistant, Pathologic Anatomy Department; Privolzhsky Research Medical University, 10/1 Minin and Pozharsky Square, Nizhny Novgorod, 603950, Russia;; Research Assistant, Medical Biophysics Laboratory, University Clinic; Privolzhsky Research Medical University, 10/1 Minin and Pozharsky Square, Nizhny Novgorod, 603950, Russia;; Research Assistant, Medical Biophysics Laboratory, University Clinic; Privolzhsky Research Medical University, 10/1 Minin and Pozharsky Square, Nizhny Novgorod, 603950, Russia;; Student; Nizhny Novgorod State Agricultural Academy, 97 Prospekt Gagarina, Nizhny Novgorod, 603107;; Researcher, Medical Biophysics Laboratory, University Clinic; Privolzhsky Research Medical University, 10/1 Minin and Pozharsky Square, Nizhny Novgorod, 603950, Russia; Researcher, Space Plasma Simulation Laboratory Federal Research Center Institute of Applied Physics of the Russian Academy of Sciences, 46 Ulyanova St., Nizhny Novgorod, 603950, Russia

**Keywords:** near-field resonance microwave sounding, dielectric properties of biological tissues, permittivity, conductivity

## Abstract

**The aim of the study** was to assess the near-field resonance microwave sounding efficiency to study the dielectric properties of investing tissues in different body areas in healthy rats.

**Materials and Methods.:**

Skin dielectric properties (permittivity and conductivity) were studied in four body parts (medial and lumbar regions of the back, forehead, abdomen) of adult Wistar rats (n=30) using near-field resonance microwave sounding. For measurements, we used a special hardware and software system designed in the Federal Research Center Institute of Applied Physics of the Russian Academy of Sciences.

**Results.:**

Dielectric properties of skin and underlying tissues significantly vary depending on a body area. The medial dorsal region was recorded to have the highest permittivity and conductivity level, while the minimum was found in the abdominal region. Frontal and caudal areas showed intermediate indices. In deepened sounding, dielectric permittivity consistently grows regardless of antenna localization (3 and 5 mm), while the conductivity recedes.

**Conclusion.:**

Near-field resonance microwave sounding enabled to reveal dielectric properties specific for each body area (both by permittivity and conductivity indices and by deep structure of their distribution). The findings should be taken into consideration in topical diagnosis of investing tissues, particularly, when assessing the wound underlying structures, the localization of wound surface boundaries, and the condition of the areas around the wound.

## Introduction

Medical imaging is one of the most dynamically developing branches of biomedicine [1–3]. The interaction of biomedical professionals, physicists, engineers, and IT specialists enables to make a noninvasive assessment diagnosis of most internal structures in humans and animals [2, 3]; however, some body parts are still uncovered by diagnostic feasibilities, and among such parts are investing tissues, the deep structure of which due to their morphological and functional characteristics is not imaged properly using modern techniques (ultrasound, computed tomography, magnetic resonance imaging, etc.) [1, 3–6]. It is due to high permittivity of the tissue for ultrasonic radiation and flat contrast for tomographic technologies.

Current diagnostic tools enable to assess only skin surface condition (dermatoscopy, IR imaging), and study just the nearest subsurface elements (e.g., optical coherent microscopy), or there are invasive techniques available (skin biopsy followed by histological examination) [1–3, 6, 7]. It determines the search for alternative methods to the existing ones. An adequate method is near-field resonance microwave sounding, which has no physical barriers for body structures, since it exhibits great penetrating depth (3 or more cm) causing no biomolecule ionization [4–9]. However, a few studies reveal the capabilities of the technology [4, 10]. Heterogeneous dielectric properties of different organs and tissue [3, 6, 8, 9] are supposed to hinder the interpretation of microwave sounding findings and require to be taken into consideration in a diagnostic study.

**The aim of the study** was to assess the near-field resonance microwave sounding efficiency to study the dielectric properties of investing tissues in different body areas in healthy rats.

## Materials and Methods

The experiment was carried out on 30 white male Wistar rats (age: 12–14 months, weight — 200–250 g) provided by the branch “Stolbovaya”, Scientific Centre for Biomedical Technology, Federal Medical and Biological Agency (Moscow). All the animals were kept in standard vivarium conditions, in cages, having free access to water and food; their diet in compliance with standards specified in GOST “Experimental animal management in research institute vivarium”. The study was carried out according to the ethic principles established by European Convention for the Protection of Vertebrate Animals used for Experimental and Other Scientific Purposes (Strasburg, 1986) and the guidelines 86/609 EEC.

Dielectric properties of skin and subcutaneous structures were studied in the frontal region of head, medial region of the back, lumbar area around the tail, as well as along the median abdominal line using near-field resonance microwave sounding. Before probing, the areas were cleaned and depilated.

**Figure 1:**
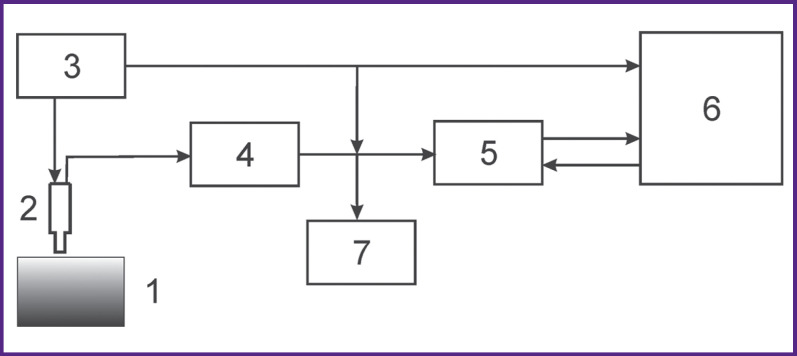
Functional block diagram for near-field microwave sounding of biological tissues in combustiology: (*1*) biological tissue; (*2*) applicator; (*3*) sweep-frequency generator; (*4*) signal detection and filtering unit; (*5*) analog-digital converter QMBOX; (*6*) personal computer; (*7*) oscillograph

Dielectric properties were assessed by a special hardware and software system (Figure 1), designed in the Federal Research Center Institute of Applied Physics of the Russian Academy of Sciences (Nizhny Novgorod), it enables to calculate dielectric permittivity and conductivity of biological tissues integrally at different probing depths [7, 8]. The present study involved the antennas to assess the mentioned characteristics at 3-and 5-mm depth. The parameters of the antennas are presented in the Table.

**Table:** Characteristics of antenna applicators for near-field microwave sounding

Indices	Probe 1	Probe 2
Resonance frequency (MHz)	540	700
Testing section radiuses (mm)	R_1_=2.5	R_1_=1.0
	R_2_=3.0	R_2_=5.0
Probing depth (mm)	3	5

The study was performed in accordance with Basel Declaration principles (2010) and the recommendations of the Ethics Committee of Privolzhsky Research Medical University.

### Statistics.

The findings were statistically processes using Statistica 6.0. The normalcy of value distribution was estimated by Shapiro–Wilk test. The findings were presented as М±σ, where М is a mean value, and σ is a mean square deviation. Student’s t-test was used to assess intergroup sampling differences. Intergroup differences were considered statistically significant if p<0.05.

## Results and Discussion

**Figure 2:**
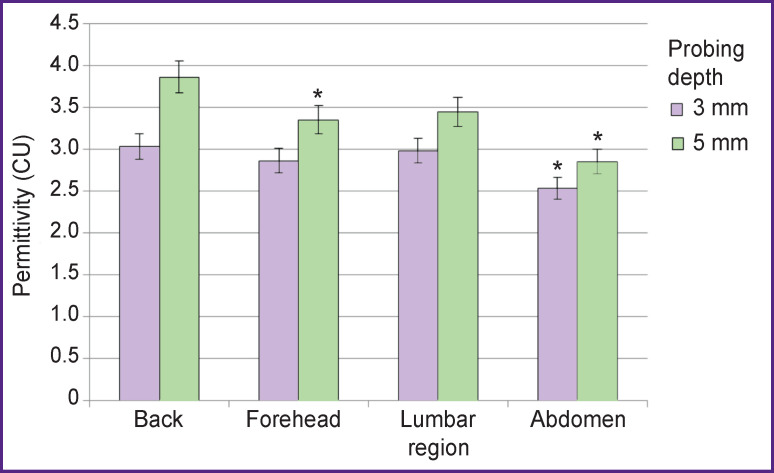
Dielectric permittivity of different rat skin areas * Statistical significance of values in reference to medial dorsal region; p<0.05

The investigations enable to find out the dielectric properties of skin and underlying tissues to vary significantly depending on the area under study. And the underlying structure of their distribution is unequal as well. Thus, medial dorsal region showed maximum real permeability (Figure 2), whereby other body areas were compared to it. It is worth noting that the regularity can be traced when probing at 3 and 5-mm depth. Lumbar caudal area (3-mm deep) of the animal back is indistinguishable by this parameter from its middle part. In contrast, the rat abdominal region demonstrated significantly lower dielectric permeability in both sounding techniques (p<0.05). Different value distribution pattern was found in the rat medial frontal region показателя: at lower depth (3 mm) it moderately differed from that of the back, while probing at 5-mm depth showed statistically significant decrease in dielectric permeability (p<0.05).

Considering the physical entity of the method due to the revealed inequality of dielectric properties, the detected differences are explained by tissue heterogeneity based on their hydration degree. The densest subsurface structures are in the back, while in probing the abdomen, the closely adjacent visceral organs can be involved, the water content of which is higher. Frontal region is somewhere between, since tissue of high hydration in the area is enclosed in rigid musculoskeletal frame that gives the correspondent distribution structure of dielectric properties.

**Figure 3:**
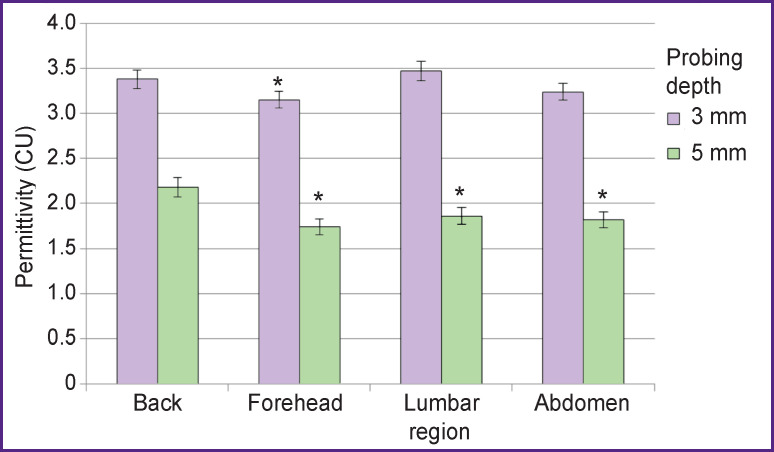
Skin conductivity in different body regions * Statistical significance of values in reference to medial dorsal region; p<0.05

The conductivity of skin and underlying tissues in all the animal body areas under study was recorded to have the opposite tendency to that revealed for dielectric permeability: with probing depth increase the conductivity decreased (Figure 3). The medial dorsal region was also found to have the highest combined (by the combination of probing depths) value of the parameter, while in the lumbar region the conductivity exceeded that typical for the back (using a measuring probe for 3-mm depth) (p<0.1), and the analysis of deeper layers showed the conductivity to be significantly lower (p<0.05). In the abdominal area, statistically significant values were recorded only when the antenna with 5-mm penetration was used (p<0.05 in reference to the medial dorsal region). Finally, the frontal area had lower conductivity when probing at both: 3 and 5 mm deep (p<0.05 for both cases).

The revealed characteristics of dielectric conductivity distribution in different body tissues are due to their heterogeneity based on hydration degree, as well as the density of adjacent tissues. The latter is the most marked in deep probing (5 mm), to a greater degree demonstrating the variability in both: conductivity and permeability of biological objects.

## Conclusion

Near-field resonance microwave sounding enabled to reveal specific dielectric properties of skin and underlying tissues of different body areas by both: conductivity and permittivity values, and also the deep structure distribution. High permittivity and conductivity is the characteristic of the medial dorsal area, while the minimal — for the abdominal region. Frontal and caudal areas rank the intermediate position. In addition, dielectric permeability consistently grows when probing depth is increased, regardless of the antenna localization; while conductivity decreases.

The study findings should be considered in topical diagnosis of different body tissues. In particular, it is of crucial importance when making proper estimate of wound underlying structures, specifying the localization of wound surface boundaries and the condition of the areas around the wound.
